# A Comprehensive Evaluation of Consumer Trends and the Bioactive Content of Extra Virgin Olive Oil: Comparative Insights into Trademarked and Local Products

**DOI:** 10.3390/foods14193384

**Published:** 2025-09-30

**Authors:** Senem Suna, Burcu Erdal

**Affiliations:** 1 Department of Food Engineering, Faculty of Agriculture, Bursa Uludag University, Gorukle, Bursa 16059, Türkiye; 2 Department of Agricultural Economics, Faculty of Agriculture, Bursa Uludag University, Gorukle, Bursa 16059, Türkiye

**Keywords:** extra virgin olive oil, sustainability, consumption trends, phenolics, antioxidant capacity, bioaccessibility

## Abstract

This multidisciplinary comparative study investigates consumption patterns, health-related properties, and quality attributes of trademarked and local extra virgin olive oil (EVOO) samples. It highlights the importance of localization in promoting agricultural sustainability, strengthening regional economies, and enhancing socio-economic impacts within EVOO production and consumption systems. In terms of quality characteristics, significant differences were observed in color parameters (L*, a*, b*, Chroma, Hue angle) among EVOO samples (*p* < 0.05). Regarding nutritional and functional properties, total phenolic content (TPC) measured with the Folin–Ciocalteu method ranged from 58.15 to 176.29 mg of gallic acid equivalents/kg of oil, while total antioxidant capacity (TAC) measured by CUPRAC and DPPH assays varied between 3.42 and 6.54 and 8.56–10.71 µmol of Trolox equivalents/g of oil, respectively. TPC and TAC were also evaluated for their stability during in vitro gastro-intestinal digestion, demonstrating that EVOO’s bioactive potential remains stable under gastric and intestinal conditions. Local samples exhibited significantly higher TACs than trademarked products across undigested, gastric, and intestinal phases (*p* < 0.05). Concurrently, a face-to-face consumer survey assessed purchasing behaviors and preferences, revealing that 71.3% of consumers preferred local EVOO and showed a low tendency to purchase commercial brands (*p* < 0.05). Cooperatives were identified as the main distribution channel, playing a crucial role in sustaining local production systems. This study offers valuable insights into EVOO’s bioactive content and consumer behavior, providing a foundation for developing both localized and commercial products that support health outcomes. Additionally, the findings contribute to policy development concerning sustainable food systems and geographical indications.

## 1. Introduction

An agriculture able to continually provide food and other resources to a growing world population is of crucial importance for human existence and hence, for any human activity. Due to this fact, sustainability in agriculture and food has become increasingly important recently. Sustainable agriculture is an “integrated system of plant and animal production practices having a site specific application that will, over the long term: satisfy human food and fiber needs; enhance environmental quality; make efficient use of non-renewable resources and on-farm resources and integrate appropriate natural biological cycles and controls; sustain the economic viability of farm operations; and enhance the quality of life for farmers and society as a whole [1,2,3]. On the other hand, being aware of environmental issues and acting positively related to these concerns, like tending to buy local products rather than choosing commercial ones, is also included in green consumerism, which is a type of consumer attitude that protects the environment [4]. Local products have positive effects in terms of sustainability by stimulating the local workforce and contributing to regional development. At this point, sustainability and sustainable development are important in programming the life and development of today and the future. It is expressed as meeting the needs of current and future generations without depleting natural resources and providing an environment for their development by establishing a balance between humans and nature [5].

As in agriculture, achieving sustainability in the olive oil sector requires a combination of many factors. Sustainability is fully achieved when it is achieved from economic, social, and environmental perspectives. In the olive sector, land and water management, reducing chemical use, protecting biodiversity, energy efficiency, and waste management are the primary environmental factors. Securing the income of olive producers, establishing long-term production plans, and developing new markets are key factors in ensuring economic sustainability for the olive and olive oil sector. Social factors such as the working conditions of olive farmers and workers, consumer awareness, and effective governance are key factors in ensuring sustainability in the olive sector [6,7,8,9].

In this concept, demand for local products plays an important role in sustainability and new technologies used extensively in the production of agriculture, food, and other basic needs have increased this demand [10,11]. “Local Products” can be defined as the products obtained from the raw material grown in its own geography and named after the region where they are produced, or the freshest and most delicious ones; whereas “Local Food” could be described as the food that was grown in close physical proximity to the consumer. Previous studies about sustainable food consumption have mentioned olive oil as a local food [12]. In addition, olive oil could be listed among the most important local products in Türkiye. Generally, olive oil production is divided into two: traditional and commercial categories. The traditional method of production plays an important role in terms of the protection and sustainability of natural resources and sensitivity to the environment while ensuring the continuity of agriculture regionally, which is an important source of income [13,14,15,16]. Production is often carried out by local businesses. These indigenous businesses are a major source of labor that revitalizes regional communities. A wide range of employment is provided at various stages such as the maintenance of olive gardens and the harvesting and production of oil, from farmers to workers and from technicians to scientists. At this point, olives directly contribute to local economies and increase the social and economic welfare of communities. Therefore, they affect the sustainability of agriculture. Herein, within the scope of sustainable agriculture, this study aimed to reveal the tendency towards local products in extra virgin olive oil (EVOO) in the context of Bursa province, Türkiye.

Olive (*Olea europea* L.) cultivation is carried out in an area of 10,338,179 hectares in the world, of which 889,168 hectares, which is 8.6%, is in Türkiye [17]. Despite domestic consumption, Türkiye is among the most influential countries in the market, with a return of USD 117 million during the year from the export of different table olive products. Even though Türkiye has suitable climatic conditions, the olive yield in the country has not yet reached the desired point, as we would like to point out. In total, 97% of the world’s olive trees are located in countries bordering the Mediterranean, including Türkiye which owns 11,389,281 fruit-bearing and 229,185 non-fruiting olive trees, respectively [18]. Additionally, in Türkiye, the olive yield per tree and the olive grain per 1 kg of olive oil is estimated to be 7.50 kg and 5.00 kg, respectively, while 2,477,220 of the specified number of trees are located in Gemlik. Since the Gemlik region is located in Bursa, this city has a very important place throughout the country In the 2023–2024 production period, a total of 2730 thousand tons of olive oil were produced in the world. Spain ranked in the first place with 780 thousand tons of olive oil production, followed by Türkiye, which comes in the second place with 380 thousand tons. Then, Türkiye is followed by Greece and Italy with 350 and 235 thousand tons, respectively [19].

The world olive oil consumption was 3329 thousand tons in the 2021–2022 product period with an increase of 4.8% compared with the previous year. A total of 46.5% of this was consumed by EU member countries. In the 2022–2023 period, this value was given as 3055 thousand tons, while the top two countries in world olive oil consumption were reported as Italy (486 thousand tons) and Spain (425 thousand tons). In Türkiye, 179 thousand tons of olive oil was consumed. Türkiye is among the Mediterranean countries where olive oil production is intense due to its location, and the importance of olives and olive oil in terms of agricultural economy in the country is great. Olive oil is an important sector with the population it employs, the added value it creates, and its competitive advantage with its high export potential.

Olive oil, one of the oldest foods in existence, is one of the distinct foods to be consumed on a regular basis because of its organoleptic and nutritional properties [20]. The most important features that distinguish olive oil from other vegetable oils are that it is obtained from olive fruit mechanically through physical processes without being subjected to any chemical treatment and has consumable qualities in its natural state.

There are many quality classes of olive oil (virgin, refined, mixed, etc.), the most important of which is extra virgin olive oil (EVOO). Factors determining EVOO quality are shaped by chemical, organoleptic, and production supply chain criteria. Among the chemical factors, the most widely known and consumer recognized parameter is free fatty acid The definition of EVOO is based on the amount of free fatty acid composition that is distinguished by the International Olive Council (2025). For example, EVOO’s unique monounsaturated fatty acid [21]. (FA) composition, which is primarily oleic acid, contributes to its high phenolic content and low acidity. Although their production processes were initially what set them apart, it has now been discovered that their molecular compositions differed as well [22]. Additionally, in 2025, the IOC established free oleic acid of ≤0.8 g/100 g for EVOO [19].

On the other hand, EVOO has become a highly demanded product in the world due to its health-beneficial effects. Those benefits are mainly attributed to fatty acids and triacylglycerols (saponifiable fractions), as well as sterols, alcohols, vitamin E, hydrocarbons, carotenoids, volatile components, and phenolic compounds (unsaponifiable fractions) The first group plays a significant role in the high stability and extended shelf life of olive oil, while the second one has drawn attention because of its antioxidative qualities and health-beneficial effects in addition to being a crucial EVOO quality characteristic.

Phenolic compounds are the main bioactive constituents of EVOO primarily com-prizing secoiridoids, lignans, phenolic acids, phenolic alcohols, and flavonoids [23,24,25]. Secoiridoids, particularly oleacein and oleocanthal—derived enzymatically from oleuropein and ligstroside—are the most abundant. Lignans (e.g., pinoresinol, 1-acetoxypinoresinol) form the second major group, while flavonoids (e.g., luteolin, apigenin), phenolic acids (e.g., caffeic, ferulic, p-coumaric, and vanillic acids) and phenolic alcohols (e.g., hydroxytyrosol and tyrosol) further contribute to EVOO’s bioactivity [26].

EVOO phenolics have anti-inflammatory, antiproliferative, antimicrobial properties, and their antioxidant activities may prevent cancer, diabetes, and neurological and cardiovascular diseases [27]. This phenolic content-related antioxidant capacity can be expressed as the starting point for many effects of EVOO. It was also noted in another study that EVOO phenolics could lower blood plasma levels of low-density lipoprotein (LDL) and stop it from oxidizing [28].

High-polyphenol EVOO has been found to provide health benefits, particularly by protecting against oxidative damage and modulating mitochondrial dysfunction [28]. They investigated the impact of varying doses of EVOO applications on the antioxidant defense status and mitochondrial activities in human keratinocyte cells and demonstrated that EVOO had a prominent antioxidant capacity under H_2_O_2_ toxicity while improved cell survival was the outcome of applying extra virgin olive oil. In another study, it was shown that using EVOO lessened the harmful effects of stress because the phenolic components in it were antioxidant molecules that might scavenge the harmful consequences of oxygen metabolism, such as the generation of free radicals, and protect cells against oxidative damage [29,30].

In a previous human study, every 10 g/2000 kcal of olive oil consumed was found to decrease the risk of all-cause, cardiovascular, and cancer mortality, lowering their ratios of 7%, 13%, and 2%. It was also determined that daily moderate consumption of EVOO (full and half tablespoons) was associated with a one-third lower risk of all-cause mortality as well as a halved risk of cardiovascular mortality [31,32].

There are several studies about extra virgin olive oil studied with diverse kinds of analysis; however, limited data about the bioaccessibility of phenolic content and antioxidative properties in EVOO have been published to date [33,34,35]. One of them also presented the chemical explanation of bioaccessibility as phenolic glycosides being hydrolyzed, leading to an increase in aglycones during the intestinal phase [36].

In this context, the issue of analyzing bioaccessible compounds found in EVOO emerges as a very current issue. Additionally, there are no studies assessing the differences in bioactives between trademarked and locally sold EVOO samples and their changes during in vitro gastro-intestinal digestion stages.

On the other hand, ensuring the sustainability of olive oil production is a necessity for consumers to have access to this product. At this point, it has led to the fact that issues such as what type of products individuals who consume olive oil prefer and how effective their nutritional content is in these preferences should be evaluated and investigated together. In this context, this study was aimed at determining consumer attitudes and behaviors by comparing local and trademarked EVOO consumption preferences in terms of the effect of health-beneficial properties on these preferences.

To the best of our knowledge, this is the first study focusing on the bioactive potential of commercial and regional EVOO samples by monitoring their changes in the gastro-intestinal tract separately, while simultaneously comparing current consumer characteristics on local and commercial products through face-to-face surveys.

## 2. Materials and Methods

This study aimed to put forth the integrity of the subject in a multidisciplinary way by evaluating the perspectives of two different branches of science together. Accordingly, it was divided into two parts. The first part aimed to determine the consumption preferences and characteristics of individuals who consumed olive oil through a face-to-face survey, while the second part aimed to conduct content laboratory analyzes of EVOO samples to reveal their beneficial effects on health. Some of the survey questions created for the study were prepared based on the information obtained after the literature review on the subject, and some were addressed taking into account the purpose and content of the study. The material of the research consisted of the data obtained as a result of the survey conducted with 272 people in order to determine the factors affecting the local olive oil consumption of individuals who consumed olive oil in the province of Bursa. In food analysis part, with the aim to reveal whether the bioactive content beneficial to health has an effect on consumer behavior, extra virgin olive oil samples were collected from a commercial trademark (EVOO1) and several local producers in the Mudanya (EVOO2), Gemlik (EVOO3), Trilye (EVOO4), and Karacabey (EVOO5) regions of Bursa, Türkiye, during the 2022 harvest season. The EVOO’s were analyzed immediately after sample collection (*n* = 3).

The survey was conducted only on individuals consuming olive oil, and since it was difficult to find a sample of the olive oil consuming group, a snowball sampling method was used. In this method, a reference person was selected regarding the subject of the study. The process was necessarily iterative. In this method, the original participants direct researchers to new participants and therefore, the sample grows; for this reason, it is referred to as the “snowball effect”.

In the snowball sampling method, the sample creation process begins by reaching one of the individuals on whom the research will be conducted. At the end of the interview with this participant, other individuals suggested by that participant are reached, and after the interview with those individuals, other individuals recommended by them are reached. Thus, the process continues with the number of participants increasing. After a while, this process ends with creating a sample by focusing on specific people [37,38]. Previously, this sampling method has been applied in several studies about food consumption preferences However, so far there is no study focusing on olive oil consumption preferences in Türkiye, and the current study will be the first study on this subject.

The main hypothesis of the study is that “An individual’s gender, age, number of people in the household, monthly income, where they buy olive oil, olive oil type, areas of use, reasons for preference, and effective factors in purchasing will significantly affect the choice of a non-trademarked (local) product”. For this purpose, a binary logistic regression method was applied to analyze the data by using the IBM SPSS Statistics package program (Version: 28.0.0.0 (190)).

Logistic regression analysis was used to model the relationship between one or more independent variables and dependent variables. At this point, the dependent variable could be categorical, and the independent variables could be continuous or categorical.

In binary logistic regression, the causality relationship between the independent variables and the dependent variable was examined, and the occurrence of the event was taken as 1 and the failure to occur was taken as 0.

Logistic regression function:

π (x) = exp (β_0_ + β_1_ x)/1 + exp (β_0_ + β_1_ x)

### 2.1. Food Analysis Methods

#### 2.1.1. Extraction of Phenolics

Initial (undigested) extracts of EVOO samples were prepared according to the previous literature [39]. For phenolics extraction, EVOO samples (1 mL) were mixed with 20 mL of water/methanol/HCl (10:80:1 *v*/*v*) mixture and shaken at 20 °C for 2 h (Memmert WNB 22, Schwabach, Germany). Subsequently centrifugation at 4 °C for 10 min (3500 rpm, Sigma3K30, Osterode am Harz, Germany) was applied before storage at −20 °C. Obtained supernatants were utilized in total phenolic content (TPC) and total antioxidant capacity (TAC) analyzes.

#### 2.1.2. In Vitro Gastro-Intestinal Digestion Procedure

EVOO samples were passed through a two-stage digestion, addressing the aliquots of gastric and intestinal extracts gathered throughout system [40]. Simulated gastric (SGF) and intestinal (SIF) fluids were arranged according to previous literature [41]. Samples were joined with SGF, porcine pepsin (25,000 U/mL, Sigma-Aldrich P6887; St. Louis, MI, USA), and CaCl_2_ along the gastric stage. Thereafter, pH was set to 3 with HCl and held at 37 °C for 2 h in a shaking water bath. Subsequently, 4 mL aliquots of each sample were gathered. The rest of the mixture was blended in with SIF, pancreatin (800 U/mL. Sigma-Aldrich P3292), and bile (160 mM) solutions individually, to mimic the intestine. A blank, containing the same reagents but excluding the food matrix, was prepared and subjected to the same experimental conditions as the samples. Afterwards, pH was adjusted to 7 with NaOH, and the chyme was shaken at 37 °C for 2 h. The chyme was then centrifuged (3500 rpm–10 min) and filtered, and the supernatant was pooled. Right after the protocol, digests were immediately cooled to 4 °C to block enzymatic reactions, then kept at −20 °C until analyzed.

#### 2.1.3. Analyses of Total Phenolics

The TPC of the undigested and digested (both gastric and intestinal digests) extracts of EVOO samples were analyzed with Folin–Ciocalteu (FC) reagent with minor modifications [42]. Concisely, deionized water/extract/FC (2.3/0.25/0.15 mL) were mixed within 15 mL volumetric flasks and vortexed (Vortex Mixer Classic, Velp Scientifica, Usmate, Italia) for 15 s at 25 ± 2 °C. Thereafter, an addition of 0.3 mL of 35% Na_2_CO_3_ was completed. Spectrophotometric absorbance of the mixtures was noted at 725 nm (Shimadzu UV-1208, Kyoto, Japan) after 2 h of incubation in the dark. Gallic acid was utilized for the calibration of the standard curve (*R*^2^ = 0.9835). Results were expressed as mg of gallic acid equivalent (GAE) per kg of oil. Since the FC reagent also reacts with interfering compounds such as ascorbic acid and sugars, the quantification of individual phenolics by HPLC–DAD/MS or LC–MS/MS techniques could be recommended.

#### 2.1.4. Determination of Total Antioxidant Capacity

Formerly prepared undigested and digested extracts were assessed for TAC. Cupric ion reducing antioxidant capacity (CUPRAC) [43] and 2,2-diphenyl-1-picrylhydrazyl (DPPH) [44] assays were conducted and Trolox was utilized for the calibration of the standard curves, respectively, given as *R*^2^ = 0.9986 and *R*^2^ = 0.9939. Total amounts were given as µmol of Trolox^®^ equivalent (TE) per g of oil.

#### 2.1.5. Determination of Bioaccessibility

Bioaccessibility was explained based on the alterations in the ratio of the samples which were subjected to gastric and intestinal stages [40]. It was evaluated in pre- and post-gastro-intestinal digestion and given as percent stability as represented in the following Equations (1) and (2).

(1) $$\mathrm{Gastric}\;\mathrm{stability}\left(\%\right)=\frac{\mathrm{TPC}\;\left(\mathrm{mg}\;\mathrm{GAE}/100g\;\mathrm{oil}\right)\;\mathrm{and}\;\mathrm{TAC}\;\left(\mu \mathrm{mol}\;\mathrm{TE}/g\;\mathrm{oil}\right)\;\mathrm{after}\;\mathrm{gastric}\;\mathrm{digestion}}{\mathrm{TPC}\;\left(\mathrm{mg}\;\mathrm{GAE}/100g\;\mathrm{oil}\right)\;\mathrm{and}\;\mathrm{TAC}\;\left(\mu \mathrm{mol}\;\mathrm{TE}/g\;\mathrm{oil}\right)\;\mathrm{before}\;\mathrm{digestion}}\times 100$$

(2) $$\mathrm{Intestinal}\;\mathrm{stability}\left(\%\right)=\frac{\mathrm{TPC}\;\left(\mathrm{mg}\frac{\mathrm{GAE}}{100g}\mathrm{oil}\right)\mathrm{and}\;\mathrm{TAC}\;\left(\mu \mathrm{mol}\frac{\mathrm{TE}}{g}\mathrm{oil}\right)\mathrm{after}\;\mathrm{intestinal}\;\mathrm{digestion}}{\mathrm{TPC}\;\left(\mathrm{mg}\frac{\mathrm{GAE}}{100g}\mathrm{oil}\right)\mathrm{and}\;\mathrm{TAC}\;\left(\mu \mathrm{mol}\frac{\mathrm{TE}}{g}\mathrm{oil}\right)\mathrm{before}\;\mathrm{digestion}}\times 100$$

#### 2.1.6. Analytical Color Features

The color of the EVOO samples was measured using a Konica Minolta CR-5 chromameter, calibrated with a standard white reference plate. The L* value indicates brightness (0 = black, 100 = white), a* shows the red–green axis (positive for red, negative for green), and b* reflects the yellow–blue axis (positive for yellow, negative for blue). Chroma (C*) measures color saturation (0 = dull, 60 = vivid), and the hue angle (h°) defines the color tone (0° = red, 90° = yellow, 180° = green, 270° = blue, 360° = red) [45].

#### 2.1.7. Statistical Analysis for EVOO Samples

A completely randomized design was managed for the estimation in triplicate. Data were assessed with the JMP program (SAS Institute-Inc., Cary, NC, USA, 27513-Version 8.0.) using one-way analysis of variance (ANOVA). F-test was utilized for analyses at the 0.05 probability level. Correlation coefficients (*R*^2^) were computed with the Excel software (Microsoft Co., Redmond, WA, USA, 2016).

## 3. Results

### 3.1. Demographic Features

Considering that demographic characteristics are important in determining consumers’ olive oil consumption, demographic characteristics such as gender, marital status, number of individuals in the household, and monthly income were examined in this study.

In total, 52.9% and 47.1% of the survey participants were men and women, respectively. The biggest group of the participants (22.4%) were between the ages of 18–29. This result showed that young people are more inclined towards local products (Table 1).

A total of 62.1% of the surveyed consumers were married, while the remaining 37.9% were single. An examination of the number of individuals in the household reveals that the biggest group (43.4%) consisted of a family of four people.

In terms of the monthly income, the highest number of people was found to be in the range of USD 300–USD 400 (Table 1). During the survey period, the average monthly income in Türkiye was around USD 480 When compared with the average, it was seen that this group was in the low-income group.

### 3.2. Extra Virgin Olive Oil (EVOO) Consumption Patterns

Data on olive oil consumption are depicted in Table 1. It was revealed that the vast majority (71.3%) preferred local products, while 28.7% chose trademarked ones. In previous research on food consumption trends, local food consumption was determined to be higher than that of commercial brands, and this was explained by taste habits inherited from the family [46]. Our results were found to be quite compatible with this study.

Among the individuals preferring trademarked products, it has been observed that consumers who procured olive oil from cooperatives (33.5%) constituted the largest group (Table 1). Cooperative was found to be the highest purchasing place. Preliminary literature also showed that consumers trusted and preferred comparative products [47].

The agricultural sales cooperatives ensure the trust of the consumer by guaranteeing the quality of the product it sells. Extra virgin olive oil (EVOO) products of “Tariş Olive” and “Olive Oil Agricultural Sales Cooperatives” continue to be marketed, sold, and distributed effectively in domestic and foreign markets in Türkiye [48]. In our study, while a large portion of consumers, 96.3%, used EVOO, the remaining 3.7% preferred “Riviera” olive oil in their diets. The fact that the majority of consumers have chosen EVOO reveals that there is a trend towards the most health-beneficial products with a good agreement with the preliminary literature [49]. Current results also supported the most important aim of this study, which was that the healthiest one should be preferred within the scope of the effects on health.

In the survey, participants reported consuming two types of olive oil, namely extra virgin olive oil (EVOO) and Riviera olive oil (Table 1). Riviera olive oil is characterized as a blend of refined and virgin olive oils, with its free fatty acid content limited to 1% (expressed as oleic acid). In this respect, Riviera represents a commercial category that is not entirely natural but positioned as a mid-range product by combining the neutral properties of refined oil with the sensory attributes of virgin olive oil. Moreover, Riviera olive oil is frequently preferred due to its milder aroma, suitability for frying, and relatively lower cost compared with other olive oil types [50]. On the other hand, regarding the usage areas of olive oil, it is possible to observe that it is mostly (56.3%) consumed in meals rather than for salads or other intended uses (Table 1). It was also thought that this situation was due to the existence of Mediterranean food culture in which olive oil consumption was of utmost importance as well as olive oil cultivation in the Marmara Region of Türkiye.

In the current study, 70.2% of consumers preferred olive oil because of its health-beneficial properties (Table 1). In great agreement with our research, a previous literature revealed that the majority of consumers (70%) preferred olive oil due to its health-beneficial properties, and 21% preferred olive oil because it was palatable and easy to digest [51]. In another research, Ağır et al. (2018) studied the reasons why consumers preferred olive oil to other oils and determined that 47.2% of them thought it was healthy, while taste and consuming habits were among the other reasons for their preference [50].

Our research also concluded that the most important criterion for consumers when purchasing olive oil was odor (48.5%), followed by color (sensorial) (30.1%), price (9.6%), sedimentation (8.1%), and packaging (3.7%), respectively (Table 1). This result showed that sensorial evaluation was more important than the cost of the product in the selection process, regardless of the financial purchasing power. In previous studies on olive oil consumption preferences, health ranked as the first among the preferences, while price ranked as the last [50].

The amount of olive oil consumption per capita in Türkiye is around 1.8 L in 2021. This amount is around 11 L in Greece and Spain, 7 L in Italy, and 6 L in Portugal [19]. The relatively reduced amount of consumption in Türkiye could be related to nutritional habits and the lack of the awareness of the health benefits of olive oil compared with other countries.

The Omnibus test, which shows the general suitability of the model, was performed, and the results are presented in Table 2.

In line with the stated purposes, a binary logistic regression model was established to determine the variables affecting the dependent variable, which was local olive oil consumption, utilizing the existing independent variables.

After all variables were added to the model, the Chi-square value was checked for model goodness of fit. It was possible to observe that the overall significance of the model, that is, the goodness of fit, was statistically significant (*p* < 0.01). *R*^2^ values showing the suitability of the model to the data and the general fit of the model are given in Table 3 and Table 4 [52].

According to the Hosmer and Lemeshow test results, the estimated logistic regression model was found to be suitable for the data (*p* = 0.617) (Table 4). Cox and Snell *R*^2^ and Nagelkerke *R*^2^ values show the magnitude of the variance explained in the dependent variable by the model. The study determined that the general fit of the model was good (Cox and Snell *R*^2^ = 0.5514; Nagelkerke *R*^2^ = 0.7890) (Table 4).

Out of the total change in consumers’ olive oil (trademarked/local) preferences, 79% was explained by the independent variables considered. This value is quite high and found statistically significant. The coefficient estimates and odds ratios of binary logistic regression analysis are presented in Table 5.

Since the logistic regression analysis was found to be significant, the Odds Exp (B) value of the coefficients in the model could be interpreted. In the age variable, under 18 years of age was accepted as the reference category. There was a positive relationship between the age variable and local olive oil use. The Exp (B) value for the Age 1 variable was found to be 73.571. Individuals between the ages of 18–29 are approximately 74 times more likely to consume local products than individuals under the age of 18. An observation of the *p* values and Exp (B) values of other age groups and a comparison of the consumption of local products with the reference category revealed that it is 50 times higher in the Age 2 category, 218 times higher in the Age 3 category, 100 times higher in the Age 4 category, and 73 times higher in the Age 5 category, respectively. This showed that local product consumption is more likely to be consumed by individuals over the age of 18.

The supermarket/delicatessen category was used as a reference to determine where consumers purchased olive oil. Those who bought from manufacturers were 3149 times more likely to use local products than those who bought from supermarkets/delicatessens. Again, an evaluation based on the reference category exhibited that the number of people who bought from the cooperative was 397 times more, those who bought from the marketplace were 168 times more, and those who had their own olive groves were 684 times more (Table 5). It was seen that the reference category, supermarket/delicatessen, was less preferred by local olive oil consumers.

Among the effective reasons for choosing olive oil, being beneficial to health was the reference category. Those who choose olive oil according to family habits preferred local products 35 times more (Table 5) because of its health benefits. Habits inherited from the family have increased the consumption of local products.

Consumers were asked what they paid attention to when buying local olive oil. In the analysis of this question, the color of the product was chosen as the reference category. Compared with color, those who paid attention to odor were four times more likely to consume local products. Additionally, the reference category revealed that the rate of local product usage by price-conscious people increased by 7.5 times (Table 5).

Monthly consumption of 250 mL was taken as the reference for the amount of olive oil consumption. The odds ratio of the monthly 1 L consumption amount category was 0.100. The adjusted odds ratio was 1/0.100 = 10. When 1 L of oil was consumed monthly, the local olive oil consumption rate decreased 10 times. The odds ratio of the monthly consumption amount category of 3 L was 0.104. The adjusted odds ratio was 1/0.104 = 9.615 (Table 5). Monthly consumption of 3 L of olive oil was 9.6 times less likely to be a local olive oil. Considering this result, consumption above the reference amount increases the probability that the product is a trademarked olive oil.

### 3.3. Total Phenolics

The total phenolic content (TPC) of EVOO samples during in vitro gastro-intestinal digestion are displayed in Table 6 (*p* < 0.05). In the current study, the highest TPC was obtained from the EVOO4 sample (186.6 mg GAE/kg oil). The TPC of the commercial sample (EVOO1) was analyzed as the lowest, with 58.1 mg of GAE/kg of oil, while local samples changed between 92.5 and 186.6 mg of GAE/kg of oil.

Korkmaz (2023) analyzed the TPC of EVOO produced from several oil varieties (Edincik, Domat, Uslu, Gemlik, Ayvalık) of Türkiye between 234.71 and 350.60 mg of GAE/kg [53]. In another study, the TPC of Turkish EVOO from different varieties (Sarı Hasebi, Gemlik, and Halkalı) and ripening stages (green, spotted, ripe) were reported between 163.02 and 749.28 mg of GAE/kg [54]. Our results were obtained from the Gemlik variety and yielded fewer results overall that might have resulted from the differences in the maturity stages of the olives used as the raw material of the EVOO. Moreover, when evaluated at the level of the final product, the observed outcomes may be attributed to storage conditions. In addition, variations in the extraction method and the compositional differences employed for the determination of bioactive compounds in this study could also have contributed to these findings [27,55].

In previous studies, the TPC of EVOO from “Frantoio” olive cultivar collected from different farms in the “Campania” region of Italy was reported as 109–250 mg of GAE/kg [56]. Baiano et al. (2013) also analyzed the TPC of EVOO in the Apulia Region of Italy as 92.9–332.3 mg of GAE/kg in order to examine the effects of genotypes and growing locations on olive oil quality [57]. Gouvinhas et al. (2014), reported the TPC of regional and commercial Portuguese olive oil between 62.77 and 219.70 mg of GAE/kg [58]. Current data (Table 6) were in line with previous reports, while the differences were related to geographical and varietal distinctions and maturity levels.

On the other hand, the choice of extraction solvent—commonly water, methanol, or acidified methanol (HCl)—significantly influences the quantification of the total phenolic content (TPC). Acidic conditions, particularly HCl, can hydrolyze esterified phenolic compounds, releasing bound phenolics that would otherwise remain undetected. While this can increase the measured TPC using the Folin–Ciocalteu assay, it may not accurately reflect the naturally occurring phenolic content in the sample. Consequently, the method may overestimate the bioavailable phenolics under physiological conditions, limiting the interpretation of TPC data when comparing extraction methods or assessing nutritional relevance. This potential overestimation should be considered when interpreting results, especially in studies linking phenolic content to antioxidant activity or health effects [59,60].

### 3.4. Total Antioxidant Capacity

Since each analysis’s measurement mechanism varies, it is necessary to evaluate multiple of them simultaneously in order to determine the total antioxidant capacity [45]. In this case, the 2,2-diphenyl-1-picrylhydrazyl radical scavenging assay (DPPH) and cupric ion reducing antioxidant capacity (CUPRAC) assay were used to measure the TAC of EVOO samples during in vitro gastro-intestinal digestion (Table 6). As stated in the TAC findings, EVOO5 and EVOO4 had the highest total antioxidant capacities in CUPRAC (6.54 μmol of TE/g of oil) and DPPH (10.71 μmol of TE/g of oil) analyses, respectively (*p* < 0.05) (Table 6).

Baiano et al. (2013) and Kesen et al. (2014) reported the DPPH analysis of EVOO and OO (olive oil) as 11.3–46.7 mg of TE/kg of oil and 0.35–0.75 mM of TE/kg, respectively [57,61], while Korkmaz (2023) reported a range between 137.91 and 515.36 mg of TE/kg in EVOO [53]. In the CUPRAC assay, 10.57 mM of TE/L was reported by Iosif et al. (2017) in OO [62]. Çelik et al. (2012) determined the CUPRAC analysis of OO methanolic extract as 0.61 µM of TE/g [63], whereas Peršurić et al. (2020) analyzed 25.74–34.49 TE/kg in an OO by-product [64]. Although not expressed in the exact units, the current findings were compatible with the literature and showed similar results. In addition, previous data varied as a result of the usage of different raw materials, formulation, and production methods. In the aspects of the TAC analyses, the commercial sample (EVOO1) had the second (5.98 μmol of TE/g of oil) and the third (10.33 μmol of TE/g of oil) highest values from the CUPRAC and DPPH assays, respectively (*p* < 0.05). This situation revealed that the commercial sample was within the analyzed limits and showed a similar trend to the local samples.

Regarding structure–activity relationships (SARs), phenolic compounds exhibit markedly different behaviors in the DPPH and CUPRAC assays. Simple phenolics such as hydroxytyrosol, with its ortho-dihydroxy (catechol) structure, show strong radical scavenging in the DPPH test owing to their high efficiency in donating hydrogen atoms and electrons. In contrast, secoiridoids, despite being structurally related to phenolic alcohols, often display weaker DPPH activity because steric hindrance and intramolecular interactions restrict their direct radical-quenching potential. In the CUPRAC method, which relies on the reduction of the Cu(II)-neocuproine complex, these bulkier and more conjugated molecules tend to respond differently, frequently exhibiting a greater or more consistent reducing capacity due to the presence of electron-donating groups and the possibility of redox cycling. Overall, SARs emphasize that molecular size, substitution patterns, and conjugation are decisive factors in shaping the assay-dependent antioxidant behavior of simple phenols compared with complex secoiridoids [65].

When the TPC and TAC analyses are evaluated all together, it was seen that the EVOO4 local sample had the highest TPC and CUPRAC values (Table 6). The commercial sample (EVOO1) resulted in having high antioxidant properties both in the CUPRAC and DPPH analyses, whereas it had been concluded to have the lowest phenolic content between the samples. Despite this difference, in general, the TPCs of the samples showed parallelism with the TAC. Correlations between the TPC and TAC were examined, and the coefficients are displayed in Table 7.

The highest correlations between the TPC and TAC were monitored from the gastric digests of the DPPH results based on the GAS DPPH-GAS TPC (*R*^2^ = 0.7233) and gastric digestion results obtained from the GAS-CUPRAC-GAS TPC (*R*^2^ = 0.6418), with partially moderate correlations, respectively. Additionally, the results of the DPPH assay based on undigested samples and gastric digests were strongly correlated with the CUPRAC of gastrically digested samples ranging from *R*^2^ = 0.9774 to *R*^2^ = 0.9847. Current findings displayed that the results of different methods used to determine antioxidant capacity were quite compatible with each other. On the other hand, negative correlations were observed in some analyses (Table 7). These opposite trends observed in certain samples were primarily associated with the changes occurring during the conditions of different stages of in vitro gastro-intestinal digestion.

A variety of methods have been developed to assess antioxidant potential from a comprehensive perspective, and several are recommended for concurrent use. The differing coefficients obtained from these approaches can be linked to the specific types of tests and origin of the samples subjected to various processing techniques, as well as the changes induced by in vitro gastro-intestinal digestion [66].

### 3.5. Effects of In Vitro Gastro-Intestinal Digestion on Phenolics and Antioxidant Capacity of EVOO

Gastro-intestinal digestion, absorption, metabolism, tissue distribution, and bioactivity are all included in the broad topic of bioavailability. It conveys the little amount of a supplement or bioactive ingredient that is consumed and finally enters the bloodstream. On the other hand, bioaccessibility, which is a term expressed within the scope of bioavailability, defines the absorption potential of the total amount of a substance. It is influenced by the nutritional model and the components that are linked with it. It changes in relation to a number of factors, such as the food’s physical characteristics, its chemical composition, and the individual’s ability for digestion [44].

It has been discovered via research on in vitro gastro-intestinal digestion that not all nutrients ingested with food are used by the body. Studies on humans and animals are required to evaluate the precise metabolic/clinical outcomes in order to support this claim. Nevertheless, determining the bioaccessibility of the targeted bioactive components by in vitro digestion is a useful and relevant approach since these sorts of in vivo research might be performed with high expenditures and long-term outcomes while necessitating ethical processes simultaneously. Herein, the currently applied standardized static in the in vitro gastro-intestinal digestion model allows for both stabilizing the experimental settings and comparing the outcomes of experimental variations.

**Figure 1:**
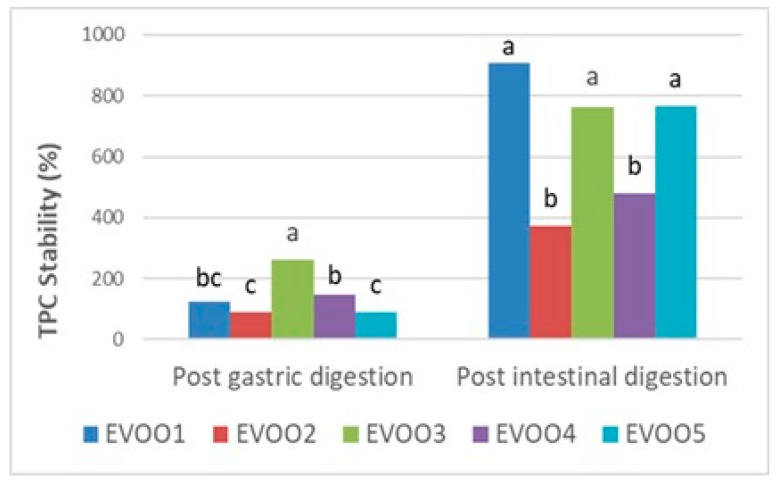
Gastric and intestinal stability (%) of TPC (total phenolic content) in extra virgin olive oil samples. The different letters a–c denote significant differences (*p* < 0.05).

**Figure 2:**
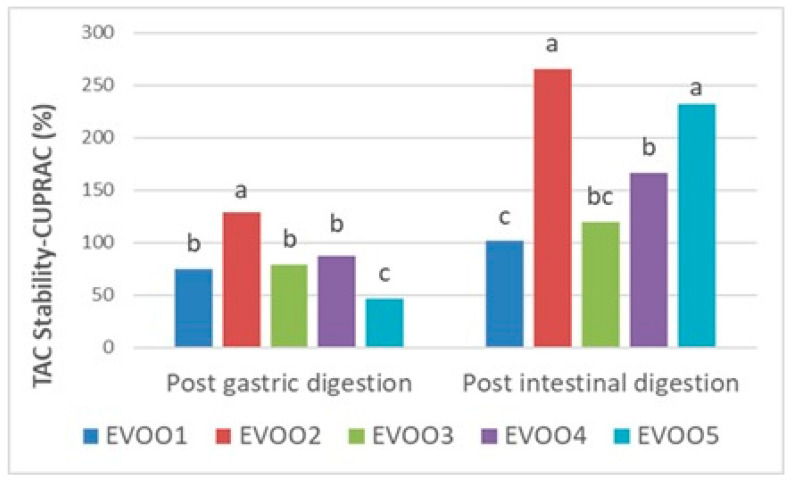
Gastric and intestinal stability (%) of TAC (total antioxidant capacity) by CUPRAC assay in extra virgin olive oil samples. The different letters a–c denote significant differences (*p* < 0.05).

**Figure 3:**
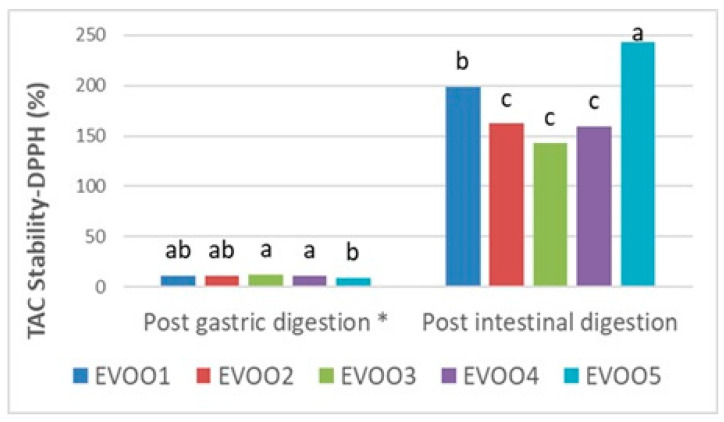
Gastric and intestinal stability of TAC (total antioxidant capacity) by DPPH assay in extra virgin olive oil samples. The different letters a–c denote significant differences (*p* < 0.05). * Post-gastric digestion of TAC Stability–DPPH (%) shows non-significance (*p* > 0.05).

Following the results at the pre- and post-digestion stages (Table 6), gastric and intestinal stability in terms of the TPC and TAC of EVOO samples are shown proportionally in Figure 1, Figure 2, and Figure 3, respectively. Phenolics from pre- and post-in vitro gastro-intestinal digestion steps were found to be statistically significant (*p* < 0.05) (Figure 1).

A significant increment in gastric (89.05–261.51%) and intestinal (373.18–906.01%) digests with respect to the undigested extracts were observed among the EVOO samples in the TPC (Figure 1) (*p* < 0.05). Especially in the post-intestinal stage, the TPC of all EVOO samples remarkably increased compared with the undigested amounts (Table 6, Figure 1) while the post-intestinal stability of phenolics was found within the literature [67,68]. Reboredo-Rodriquez et al. (2021) also used the same in vitro digestion method as in our study and reported a similar increase in post-gastric and post-intestinal conditions of phenolics, which is quite consistent with our results [69]. This increment was also reported as the chemical transformation of phenolic compounds as a consequence of gastro-intestinal digestion. Additionally, this is likely attributable to the hydrolysis of bound phenolic compounds into smaller derivatives, such as hydroxytyrosol, tyrosol, oleuropein, hydroxycinnamicacids, and flavonoids [70].

The bioaccessibility of phenolics in food depends on the composition of the food and the applied processes. This was attributed to the fact that when exposed to gastro-intestinal stages, polymerized polyphenolics hydrolyzed into monomers and aglycons, increasing the total amount of free phenolics available [66,71] Moreover, the release of phenolics from the food matrix is influenced by digestive enzymes and pH changes between different stages of digestion [45].

In the CUPRAC assay, a mild decrease after gastric digestion resulting with stability between 46.92 and 128.84% was reported in comparison with the undigested extracts (*p* < 0.05). In this range, EVOO2 resulted in being the most stable one to gastric conditions than the other samples. Afterwards, all samples showed an increasing trend in the post-intestinal digestion stage with quite high stabilities (101.43–266.00%) (Figure 2). EVOO2 was again the sample whose antioxidative properties were more compatible with intestinal conditions and showed the best stability (266.00%) in this environment.

On the other hand, the TAC of all the samples increased after intestinal digestion compared with the initial amounts in the CUPRAC assay (*p* < 0.05) (Table 6; Figure 2). This increment between the pre- and post-intestinal digestion of EVOO samples, and additionally the post-digestion increasing trend in the CUPRAC was found to be similar to the former literature [72].

Similarly to the CUPRAC results, an increment (142.86–243.90%) (*p* < 0.05) in the post-intestinal digests following the decrement, which resulted in 9.11–11.98% stability in the pre-intestinal stage, was analyzed in comparison to initial extracts in the DPPH method. This outcome could be linked to the degradation of unstable phenolics, particularly oleuropein aglycone, as a result of the overall decrement in gastric digestion [73].

In terms of the TAC, although there was an increase in the pre-intestinal digestion phase, this was not found to be statistically significant (*p* > 0.05) (Figure 3). Furthermore, the highest antioxidative stability ratios were obtained from EVOO3 and EVOO5 at the pre-and post-intestinal phases, respectively (Figure 3). According to the current results, it has been shown that in post-gastric and post-intestinal conditions, the TAC stability increment ratios were quite similar in both assays despite the fact that the TPC showed a considerable increase, especially after post-intestinal digestion. This could be explained by the fact that phenolics in EVOO samples are more resistant to intestinal conditions in the TPC method than in TAC analysis mechanisms. On the other hand, at the post-intestinal stage, the TAC (CUPRAC, DPPH) results of EVOO samples all increased compared with the initial extracts, proving the positive correlation with the phenolic content trends [74].

Structure of the food matrix, tissue disruption and antioxidant release, conversion of phenolics with antioxidative qualities into other components, and the chemical basis of antioxidant capacity determination techniques all influence how gastro-intestinal digestion affects antioxidant properties. Furthermore, the degradation of phenolics into smaller molecular components after colonization of the human gut microbiota facilitates their full absorption [75]. In light of this information, current findings corroborate the idea that bioaccessible phenolics rise throughout intestinal digestion.

### 3.6. Analytical Color Results

Color is a critical aspect of food quality, often serving as the primary criterion by which consumers assess products and make purchasing decisions. Beyond its role in initial evaluation, color significantly influences consumer preferences and perceptions. There are multiple factors affecting the color attributes of EVOO samples, ranging from quality of the raw olives and processing methods to the packaging and storage conditions. The present study seeks to determine whether these color characteristics, which mainly resulted from chlorophyll and carotenoid content in the sample, influence consumer perceptions and preferences.

The analytical color features of the EVOO samples are displayed in Table 8. The L* values exhibited significant differences (*p* < 0.05), ranging from 26.86 ± 0.80 to 31.63 ± 0.04 (Table 8). In comparison, previous studies reported L* values as follows: 22.92–25.59 by Ocakoğlu et al. (2009) [76], 66.21–67.24 by Lolis et al. (2020) [77], 70.81 by Al Juhaimi et al. (2016) [78], and 90.83 by Ayadi et al. (2009) [79]. The L* values obtained in the present study fall within the broader range reported in the literature, which can primarily be attributed to differences in olive genotype, harvest time, and geographical origin.

The a* values of the EVOO samples were found to range between −2.80 ± 0.05 and −1.74 ± 0.01 (Table 8). These results are in agreement with those reported by Ocakoğlu et al. (2009), who documented values between −1.97 and −1.15 [76]. Similarly, the findings are comparable to those reported by Al Juhaimi et al. (2016) (−3.69), Lolis et al. (2020) (−6.62 to −5.24), and Ayadi et al. (2009) (−13.00), although some of these were lower values [77,78,79].

The b* values of the EVOO samples, ranging from 5.54 ± 0.06 to 11.03 ± 0.27 (Table 8), were consistent with the findings of Ocakoglu et al. (2009), who reported values between 10.67 and 13.76 [76]. However, these values were notably lower than those reported by Al Juhaimi et al. (2016) and Ayadi et al. (2009), who found b* values of 38.26 and 68.87, respectively [78,79].

The Chroma value (C*), representing the intensity of color perception, ranged from 5.81 ± 0.06 to 11.38 ± 0.28 with EVOO4 exhibiting a significantly higher value (11.38 ± 0.28) compared with others (*p* < 0.05). The hue angle (h°) of the samples was also found to be statistically significant (*p* < 0.05), ranging from 80.24 ± 0.10 (EVOO3) to 84.97 ± 0.12 (EVOO4). In accordance with the Chroma values, the hue angle (h°), which defines the color tone, was found to be highest in EVOO4 (Table 8).

Considering all color metrics comprehensively, EVOO3 and EVOO4 exhibited the most favorable color characteristics among the samples. In this context, analytical color measurements demonstrated that the local samples exhibited significantly enhanced color characteristics relative to the commercial sample.

## 4. Discussion

Since food sustainability is an actual issue that needs to be evaluated with a multidisciplinary approach, the current characteristics of consumers and the nutritional properties of EVOO are presented in terms of economic and bioactive potential, respectively. In both approaches, factors affecting trademarked and local product preferences in EVOO were evaluated.

EVOO is a highly demanded product with several of its health promoting effects mainly based on polyphenolic and antioxidant contents. These components are the factors that strengthen and reveal the health-beneficial effects of EVOO. Therefore, once the antioxidant capacity and total phenolic content data are available, the contribution of EVOO consumption to the daily intake and right after their progression in the gastro-intestinal system could be calculated.

However, determining only bioactive potential or current production/consumption data does not reveal how health-promoting components affect consumer behavior, and unfortunately they remain as two separate main topics. At this point, the aim of this study was to determine which of the commercial and local products was consumed more and then to analyze the bioactive potential and bioaccessibility of these products to reveal whether their beneficial effects on health are in line with consumer preferences. Although phenolic compounds are not directly detectable by consumers, they are associated with sensory attributes such as bitterness and pungency. For instance, secoiridoid phenolics, including oleocanthal and oleacein, are primarily responsible for these sensory characteristics, thereby shaping consumer preferences.

In light of the agricultural economics perspective, local and trademarked olive oil consumption was examined through a survey. Consumption of local olive oil products was higher than commercial/trademarked branded olive oil, and the product preference was extra virgin olive oil (EVOO) which was also the type of product we investigated to be the most preferred due to its beneficial effects on health. The primary factor of choice was that the product was perceived as beneficial to health. The logistic regression results revealed the existence of a statistically positive relationship between age and local olive oil consumption. Consumption of local products increased depending on age. It has also been determined that consumers who care about odor tend to prefer local olive oil when consuming olive oil.

Current knowledge could be used by policy makers to plan actions in support of sustainability and its enhancement for the success of local businesses. At this point, knowing the consumers’ opinions about the sustainability of this local product is very important in terms of evaluating how local production could be developed.

Herein, it was also expected to support the idea that locally produced foods may have higher bioactive potential and a healthier composition than those sold commercially, which could encourage local production. Consequently, bioactive and bioaccessible results of EVOO revealed that local samples exerted higher results when compared with the trademarked one with regard to antioxidative characteristics (TPC, DPPH, and CUPRAC analysis) in undigested, gastrically digested, and intestinally digested phenolics.

Moreover, our results have put forth that EVOO had nutritive effects as well as exerted good stability under gastric and intestinal conditions in the digestive track. These findings were further corroborated by analytical color measurements, considered one of the most important quality indexes, which demonstrated that local samples exhibited superior color characteristics compared with the commercial sample.

This study was conducted in a single region that is a leader in EVOO production. As the study design was based on this, it would be beneficial to cover all geographical regions and thus evaluate the data of a wider audience and repeat it with a larger sample to support the results. Therefore, direct and generalized comments were avoided. In conclusion, this pioneering study will serve as an important example for future studies aimed at suggesting consumption patterns and evaluating local/commercially available EVOO formulations that may be responsible for the observed beneficial health effects and will shed light on stakeholders working on the subject of geographical indication.

## Figures and Tables

**Table 1:** Demographic characteristics.

Demographic Characteristics	*Frequency*	*%*
Female	128	47.1
Male	144	52.9
Age		
Under 18	8	2.9
18–29	61	22.4
30–39	52	19.1
40–49	50	18.4
50–59	55	20.2
60 and over	46	16.9
Marital Status		
Married	169	62.1
Single	103	37.9
Number of Individuals in the Household		
1	17	6.3
2	53	19.5
3	83	30.5
4	118	43.4
5 and over	1	0.4
Monthly Income		
USD 300–400	83	30.5
USD 401–501	62	22.8
USD 502–602	78	28.7
USD 603 and over	49	18.0
Product Preference		
Trademarked	78	28.7
Non-trademarked (local)	194	71.3
Purchasing Place		
Supermarket/Delicatessen	61	22.4
Producer	59	21.7
Cooperative	91	33.5
Marketplace	11	4.0
Factory	2	0.7
Owns Olive Grove	48	17.6
Olive Oil Type		
Extra Virgin Olive Oil (EVOO)	262	96.3
Riviera Olive Oil	10	3.7
Area of Use		
Salad	97	35.7
Food	153	56.3
Other	22	8.1
Reason for Preference		
It is Healthy	191	70.2
It is Delicious	30	11.0
Habit from the Family	27	9.9
High Nutritional Value	18	6.6
Shelf Life	6	2.2
Factors Considered While Purchasing		
Color	82	30.1
Odor	132	48.5
Sediment	22	8.1
Price	26	9.6
Package	10	3.7
Amount		
250 mL	29	107
500 mL	45	16.5
1 L	78	28.7
2 L	85	31.3
3 L and above	35	12.9

**Table 2:** Omnibus tests of model coefficients.

		Chi-Square	df	Sig.
Step 1	Step	217.711	36	<0.001
	Block	217.711	36	<0.001
	Model	217.711	36	<0.001

**Table 3:** Hosmer and Lemeshow test.

Step	Chi-Square	Df	Sig.
1	6.271	8	0.617

**Table 4:** Model summary.

Step	−2 Log Likelihood	Cox & Snell *R* Square	Nagelkerke *R* Square
1	108.269	0.551	0.789

**Table 5:** Coefficient estimates and odds ratios of binary logistic regression.

Independent Variables	B	Significance	Exp (B)
Gender 1	−0.284	0.663	0.753
Age (1)	4.298	0.027 *	73.571
Age 2	3.914	0.073 **	50.089
Age 3	5.358	0.024 *	218.064
Age 4	4.607	0.041 *	100.204
Age 5	4.285	0.063 **	72.609
Marital Status 1	−0.725	0.500	0.484
Household 1	2.146	0.192	8.554
Household 2	1.163	0.445	3.200
Household 3	2.078	0.175	7.986
Household 4	−19.872	1.000	0.000
Monthly Income 1	−0.791	0.337	0.454
Monthly Income 2	−0.067	0.935	0.935
Monthly Income 3	1.520	0.174	4.573
Place 1	8.055	<0.001 *	3149.039
Place 2	5.984	<0.001*	396.834
Place 3	5.125	<0.001 *	168.092
Place 4	23.407	0.999	146.240
Place 5	6.528	<0.001 *	683.869
Olive Oil Type 1	−0.053	0.937	0.949
Olive Oil Type 2	−2.329	0.230	0.097
Area of Use 1	0.653	0.371	1.921
Area of Use 2	−0.087	0.941	0.916
Preference 1	1.555	0.160	4.734
Preference 2	3.570	0.056 **	35.503
Preference 3	0.781	0.607	2.184
Preference 4	−1.772	0.280	0.170
Purchase 1	1.419	0.028 *	4.134
Purchase 2	1.153	0.311	3.169
Purchase 3	2.027	0.068 **	7.588
Purchase 4	−17.705	0.999	0.000
Amount 1	−1.981	0.127	0.138
Amount 2	−2.298	0.075 **	0.100
Amount 3	−2.266	0.094 **	0.104
Amount 4	−0.939	0.539	0.391
Constant	−1.806	1.000	0.164

(*) Significant at 5% significance level; (**) significant at 10% significance level.

**Table 6:** Data were expressed as the mean ± standard deviation (*n* = 3). Different lower-case letters in the column are significantly different (*p* < 0.05). TPC was expressed as milligrams of gallic acid equivalent (GAE) per 100 g of oil and TAC results were given as micromole of Trolox equivalent (TE) per gram of oil. TACs were analyzed with CUPRAC and DPPH methods. Regions of extra virgin olive oils; EVOO1: Commercial brand, EVOO2: Mudanya, EVOO3: Gemlik, EVOO4: Trilye, and EVOO5: Karacabey.

Analysis	Undigested (Initial)	After In Vitro Digestion Process	
		Gastric Digestion	Intestinal Digestion
**TPC (mg GAE/kg oil)**			
**EVOO1**	58.15 ± 1.39 ^c^	71.47 ± 1.78 ^d^	605.01 ± 5.63 ^b^
**EVOO2**	176.29 ± 2.19 ^a^	159.15 ± 0.91 ^c^	646.33 ± 2.31 ^b^
**EVOO3**	92.51 ± 1.60 ^bc^	235.94 ± 0.75 ^b^	690.12 ± 2.29 ^b^
**EVOO4**	186.62 ± 2.21 ^a^	273.00 ± 1.13 ^a^	890.50 ± 5.16 ^a^
**EVOO5**	101.16 ± 2.35 ^b^	87.27 ± 0.13 ^d^	750.92 ± 1.92 ^ab^
**TAC (μmol TE/g oil)** **CUPRAC**			
**EVOO1**	5.98 ± 0.18 ^a^	4.45 ± 0.69 ^a^	6.07 ± 0.23 ^c^
EVOO2	3.42 ± 0.80 ^b^	4.31 ± 0.36 ^a^	10.46 ± 0.79 ^b^
EVOO3	5.73 ± 0.58 ^a^	4.53 ± 0.74 ^a^	6.80 ± 0.43 ^c^
EVOO4	5.51 ± 0.37 ^a^	4.83 ± 0.55 ^a^	9.15 ± 0.76 ^b^
EVOO5	6.54 ± 0.79 ^a^	3.06 ± 0.33 ^b^	14.88 ± 0.79 ^a^
**DPPH**			
EVOO1	10.33 ± 0.36 ^a^	1.10 ± 0.24 ^a^	20.53 ± 1.47 ^a^
EVOO2	10.40 ± 0.04 ^a^	1.14 ± 0.08 ^a^	16.87 ± 0.10 ^b^
EVOO3	10.29 ± 0.46 ^a^	1.22 ± 0.03 ^a^	14.75 ± 2.48 ^b^
EVOO4	10.71 ± 0.57 ^a^	1.23 ± 0.10 ^a^	17.07 ± 1.33 ^b^
EVOO5	8.56 ± 0.19 ^b^	0.78 ± 0.02 ^b^	20.88 ± 2.14 ^a^

**Table 7:** Pearson’s correlation coefficients between TPC, TAC, and in vitro gastro-intestinal digestion procedure.

	Correlation Coefficient (*R*^2^)								
Analyses	UND TPC	GAS TPC	INT TPC	UND CUPRAC	GAS CUPRAC	INT CUPRAC	UND DPPH	GAS DPPH	INT DPPH
UND TPC	1.0000								
GAS TPC	0.6126	1.0000							
INT TPC	0.5874	0.6350	1.0000						
UND CUPRAC	0.6426	−0.2272	0.2339	1.0000					
GAS CUPRAC	0.5874	0.6418	0.0856	−0.3684	1.0000				
INT CUPRAC	0.2761	−0.2673	0.3277	0.0791	−0.8337	1.0000			
UND DPPH	0.3548	0.5803	0.0038	−0.5114	0.9847	−0.7963	1.0000		
GAS DPPH	0.3341	0.7233	0.0689	−0.4444	0.9774	−0.7978	0.9652	1.0000	
INT DPPH	−0.4225	−0.8446	−0.1543	0.4661	−0.6193	0.4047	−0.6013	−0.7677	1.0000

CUPRAC and DPPH assays were used in the determination of total antioxidant capacity (TAC). UND, undigested; GAS, gastric digestion; INT, intestinal digestion; TPC, total phenolic content.

**Table 8:** Data were expressed as the mean ± standard deviation (*n* = 3). Different lower-case letters in the column are significantly different (*p* < 0.05). Regions of extra virgin olive oils; EVOO1: Commercial brand, EVOO2: Mudanya, EVOO3: Gemlik, EVOO4: Trilye, and EVOO5: Karacabey.

Samples	L*	a*	b*	Chroma (C*)	Hue Angle (h°)
EVOO1	30.67 ± 0.61 ^ab^	−1.82 ± 0.05 ^a^	5.86 ± 0.15 ^d^	6.14 ± 0.16 ^d^	80.75 ± 0.23 ^d^
EVOO2	30.87 ± 0.63 ^ab^	−2.36 ± 0.04 ^c^	8.46 ± 0.20 ^b^	8.78 ± 0.20 ^b^	83.50 ± 0.15 ^b^
EVOO3	31.63 ± 0.04 ^a^	−1.74 ± 0.01 ^a^	5.54 ± 0.06 ^d^	5.81 ± 0.06 ^d^	80.24 ± 0.10 ^e^
EVOO4	29.89 ± 0.15 ^b^	−2.80 ± 0.05 ^d^	11.03 ± 0.27 ^a^	11.38 ± 0.28 ^a^	84.97 ± 0.12 ^a^
EVOO5	26.86 ± 0.80 ^c^	−2.10 ± 0.07 ^b^	7.64 ± 0.38 ^c^	7.93 ± 0.34 ^c^	82.80 ± 0.31 ^c^

## Data Availability

The original contributions presented in the study are included in the article, further inquiries can be directed at the corresponding author.
